# Editorial

**Published:** 1980

**Authors:** 


### Editorial

This Journal approaches its Centenary in 1983 in a state of convalescence. Inordinate delays and difficulties have at last been solved by a change of printer and now after two such changes in 5 years we are in the hands of the University of Bristol Printing Unit. We may hope that troubles on this score are over.

Since 1975 however, this quarterly journal has produced only two editions each year and has lost its advertising revenue. Moreover, it is more than a year in arrear.

The question has to be faced – Is there now a need for a journal such as ours? In addition to the British Medical Journal and the Lancet, which doubtless most members receive along with the Practitioner or other journal appropriate to their speciality, we are bombarded with 'free' journals. Some of these, like the British Journal of Hospital Medicine are of high quality, Hospital Update is beautifully produced, there is much racy reading in World Medicine. The BMA News Review arrives regularly through our letter boxes, so does Health Trends, Prescriber's Journal, Drugs and Therapeutics Bulletin, to name only a few. Annals come from the various Colleges, annual reports from the Medical Defence Union and from the General Medical Council, many of us take the Medical Annual, the list seems endless. It is impossible to read it all. So why not let our journal faced with this plethora of print, simply fade away and retire honorably, after a fine innings of nearly a century?

Apart from the pain of allowing an old friend, who can be saved with a little effort, to die, your editorial committee believes that there is as important a place as ever for a local medical magazine. Bristol is a great medical centre in a great city. The journal of the Bristol Medical School – The Black Bag, is no more. The Bristol Medico-Chirurgical Journal is our one surviving voice and our only archive. It is also one of the oldest medical journals in existence, its publications are listed in the Index Medicus. It finds its way into Libraries throughout the world. The University Library receives a number of other journals in exchange for ours. Your new editor finds himself in his present position because of his conviction that papers given at the specialist meetings in the South West are of a high order and deserve publication. As a result papers given at the meetings of the South West Surgical Club are now appearing in abstract in this journal. Abstracts have now been received from the South West Orthopaedic Club, it is hoped that the Physicians and other specialist Societies will also send us material. In this way a valuable archive of the proceedings of these societies will be built up.

The main and most important function of this journal is to publish papers given to our own society and the Long Fox lectures. Many important speakers address us, and to browse through back numbers is to be constantly surprised by the quality of these papers and the range of subjects. Most consistently interesting are the Presidential addresses, usually the distillation of a life's work or a main interest of Bristol's most distinguished doctors.

Other medical societies besides our own flourish in this city, – The Cossham Medical Society, The Bristol Medico-Legal Society, The Bristol Medical Group, valuable papers are given at their meetings as well as at the regular Postgraduate Meetings at the Royal Infirmary, at Southmead and at Frenchay hospitals. We should aim to gather in this material as well. There is still also, as there has always been an important place for original contributions whether of work done, recollections of people or of places visited or historical studies. Records of local history have a particular place in a journal such as ours and perhaps their only place. Obituaries must be written, Books by local authors should be reviewed.

The material is there to be harvested and the result could be a journal which no Bristol doctor would wish to be without and which advertisers would be glad to patronise.

If, during 1982 we can produce four half-yearly editions, we can enter 1983, our Centenary year publishing on time. We can then expect to find advertisers and to achieve financial as well as literary health. Meanwhile 1982 will be an uphill struggle, transfusion will be needed.

